# Prevention of Depression and Anxiety in Subclinical Adolescents: Effects of a Transdiagnostic Internet-Delivered CBT Program

**DOI:** 10.3390/ijerph19095365

**Published:** 2022-04-28

**Authors:** Julia C. Schmitt, Rosa M. Valiente, Julia García-Escalera, Sandra Arnáez, Victoria Espinosa, Bonifacio Sandín, Paloma Chorot

**Affiliations:** Departamento de Personalidad, Evaluación y Tratamientos Psicológicos, Facultad de Psicología, Universidad Nacional de Educación a Distancia (UNED), 28040 Madrid, Spain; rmvalien@psi.uned.es (R.M.V.); jgarciaescalera@psi.uned.es (J.G.-E.); sarnaez@bec.uned.es (S.A.); vespinosa36@alumno.uned.es (V.E.); bsandin@psi.uned.es (B.S.); pchorot@psi.uned.es (P.C.)

**Keywords:** transdiagnostic, AMTE, anxiety, depression, feasibility, emotion regulation, adolescents, indicated prevention, internet-delivered CBT, UP-A

## Abstract

Anxiety and depressive symptoms are common problems in adolescence that could be addressed by means of preventive interventions. Even though transdiagnostic cognitive behavior therapy (T-CBT) is potentially an ideal strategy to deal with anxiety and depression, it has rarely been used for preventive purposes. In addition, so far, no study has used internet-delivered T-CBT to prevent anxiety and depression in adolescents. This study aimed to examine the utility of AMTE, an internet-delivered T-CBT program, for the indicated prevention of anxiety and depression in adolescents. AMTE was applied to 30 adolescents (56.7% females, age range = 12–18 years, *M*_age_ = 14.00, *SD*_age_ = 1.89) who showed subclinical symptoms of anxiety and/or depression. Participants were assessed at pre- and post-treatment and follow-up (3 months). We found that after the program, the symptoms of self-reported anxiety and depression, clinician-rated symptom severity, and self-reported and parent-reported severity of the main problems had significantly improved. In addition, there were significant improvements in anxiety sensitivity and emotional avoidance. Finally, we found high feasibility and acceptability of the program. AMTE is feasible and potentially effective for the indicated prevention of anxiety and depression as well as of clinical transdiagnostic factors, in adolescents.

## 1. Introduction

Recent studies on the psychological impact of the COVID-19 pandemic suggest that, among adults, anxiety and depression are more prevalent in younger people [1,2]. In addition, anxiety and depression have been very prevalent in children and adolescents during the COVID-19 pandemic [3]. In general, prevalence rates of anxiety and depressive disorders and subclinical symptoms in adolescents are high. Kessler et al. [4] found lifetime prevalence rates of 32.4% for anxiety disorders and 10.6% for depression in adolescents (aged 13 to 17), and Balász et al. [5] revealed in their study that 32% of adolescents showed subclinical symptoms of anxiety and 29.2% subclinical symptoms of depression. Anxiety and depression can have serious mental health, social, and academic consequences for the adolescents concerned [5,6]. Many adolescents who suffer from anxiety or depressive disorders or symptoms also show comorbid disorders or symptoms of the other disorder. For example, Axelson and Birmaher [7] reported that 10–15% of children and adolescents with an anxiety disorder also have depression, and 25–50% of those who have depression also have an anxiety disorder. In adolescents, reporting anxiety or depressive symptoms is strongly associated with reporting symptoms of the other condition as well [5].

Given their early onset and negative consequences, a focus on the prevention of these disorders seems desirable. Mrazek and Haggerty [8] distinguished three forms of prevention for mental disorders, that is, universal, selective, and indicated prevention. While universal prevention targets all individuals without taking into consideration their risk for developing a disorder, selective prevention approaches individuals who present a social, biological, or psychological risk factor for developing a certain disorder, and indicated prevention addresses individuals who show a biomarker associated with the risk of developing a certain disorder or elevated symptoms of the disorder but no diagnosis. Most of the anxiety and depression prevention programs for children and adolescents are administered in a school setting [9]. An advantage of this setting certainly is the reachability of the target group, and that school hours and premises are used to select the sample and/or to administer the programs. In their systematic review and meta-analysis, Werner-Seidler et al. [10] identified a total of 118 unique randomized controlled trials (RCTs) and included 108 in the meta-analysis on psychological or psycho-educational anxiety and depression prevention programs administered in schools (91.77% of the programs were based on cognitive behavior therapy; CBT), of which only 39 studies were focused on indicated prevention, and just 30 targeted both anxiety and depressive symptoms (of these, only 3 studies applied indicated prevention using a sample of adolescents). Overall, psychological prevention programs reduced anxiety and depressive symptoms in children and adolescents, showing small effects. Selective and indicated prevention for depression produced greater effects than universal prevention.

Transdiagnostic CBT (T-CBT) targets comorbidity by treating common symptoms and risk and maintaining factors of groups of disorders [11] and has shown to be effective in treating comorbid anxiety and depressive disorders [12]. Despite the potential benefits of T-CBT preventive interventions, few transdiagnostic studies have simultaneously addressed the prevention of anxiety and depression. Applying the Unified Protocol for Transdiagnostic Treatment of Emotional Disorders in Adolescents (UP-A) [13] (for the Spanish translation and adaptation, see Ehrenreich-May et al. [14]), some authors provided preliminary evidence on its utility as a universal prevention tool for anxiety and depression in adolescents. The study of Ehrenreich-May and Bilek [15] found significant decreases in anxiety from pre- to post-intervention but no changes in depression in a sample of children aged 7–10 years. Using a Spanish adaptation of the UP-A as a school-based universal prevention program [16], an uncontrolled study (*n* = 28) conducted by García-Escalera et al. [17] showed that participation in the program was associated with significant declines in self-reported anxiety symptoms, interference of anxiety and depression, and top problems’ mean severity. Additionally, in a cluster RCT with a three-month follow-up, no differences were found between the universal adaptation of the UP-A and a wait-list control group, but exploratory analyses revealed that adolescents with greater baseline emotional symptoms in the UP-A group trended towards a significantly greater decrease in depressive symptoms compared to the wait-list control group, warranting the evaluation of the UP-A adapted as an indicated prevention program [18]. According to the meta-analysis of Werner-Seidler et al. [10], no study has applied a transdiagnostic protocol for the indicated prevention of anxiety and depression in adolescents. The recent study by Ramdhonee-Dowlot et al. [19] revealed that the Super Skills for Life program [20,21] is effective to improve both anxiety and depressive symptoms in children and adolescents in residential care institutions; however, the authors did not report separate data for the adolescent sample. To our knowledge, no study has yet examined the effects of this program when applied to adolescents with symptoms of anxiety and depression.

The UP-A may be particularly well suited as a prevention program for various reasons, including the following: (a) the program is a manualized protocol that has provided support mitigating symptoms of both anxiety and depression in children and adolescents [12,22,23,24]; (b) it focuses on emotion regulation to prevent a wide range of symptoms because deficits in emotion regulation in youth have been found to play a role in the development and maintenance of several emotional disorders, including anxiety and depression [25,26]; and (c) the UP-A has a flexible nature, which, along with a reduced number of core modules (eight), makes this treatment protocol a good candidate to be easily adapted as a prevention program [18].

Internet-delivered CBT (iCBT) interventions represent an emerging way to improve access to evidence-based treatments for anxiety and depression. Internet-based treatments have several advantages compared with traditional face-to-face interventions, including better cost-effectiveness, reduced stigma, higher temporal and geographical accessibility, and general availability [27]. Likewise, participants in internet interventions may receive therapist support faster than getting access to traditional psychotherapy. Several reviews and meta-analyses have shown comparable reductions in internalizing symptoms after iCBT and face-to-face CBT in adults, children, and adolescents [12,28,29,30,31]. 

Recently, Sandín et al. [32] developed AMTE (Aprende a Manejar tus Emociones; Learn to Manage your Emotions), an online program based on the UP-A. To the best of our knowledge, AMTE is the first transdiagnostic, internet-delivered intervention program designed to manage anxiety and depressive symptoms in adolescents. AMTE focuses on strengthening emotion regulation strategies, follows a flexible modular approach, is self-administered, includes the telephone support of a therapist, and involves parents or caregivers in the program.

A study based on a sample of adolescents mainly with comorbid anxiety and depressive disorders (*n* = 12) revealed that AMTE is feasible and acceptable according to the participants and their parents and provided preliminary evidence of its clinical utility to improve internalizing symptoms in adolescents, specifically anxiety and depression symptoms, panic disorder symptoms, panic disorder severity, generalized anxiety disorder symptoms, pathological worry, and major depressive disorder symptoms [33]. Furthermore, the authors found significant reductions in transdiagnostic vulnerability factors, including anxiety sensitivity and emotional avoidance. The recent study (*n* = 15) by Păsărelu et al. [34] provides support for the clinical utility of a transdiagnostic internet-delivered rational emotive intervention for adolescents with anxiety and depressive disorders.

The present study aimed to preliminarily test the utility of AMTE for the prevention of depression and anxiety in a sample of subclinical adolescents in Spain. As far as we know, this is the first study to investigate the effects of a prevention program for anxiety and depression in adolescents based on transdiagnostic iCBT. The sample comprised adolescents with increased depression and/or anxiety symptoms but without a psychological disorder to clearly evaluate the use of AMTE for the indicated prevention of depression and anxiety. Based on recent promising findings concerning AMTE [33] and on our results regarding the UP-A as a universal prevention program for anxiety and depressive symptoms in adolescents in a school setting [17,18], we hypothesized improvements from pre-treatment to post-treatment and follow-up in (a) self-reported anxiety and depressive symptoms as well as in clinician-rated symptom severity of anxiety and depressive symptomatology (first hypothesis); (b) self-reported positive and negative affect, anxiety sensitivity, and emotional avoidance (second hypothesis); and (c) top emotional problem severity rated by the adolescent and the parent (third hypothesis). According to a fourth hypothesis, we expected to find that the internet-delivered intervention would be feasible and acceptable for adolescents with subclinical symptoms of anxiety and depression.

## 2. Materials and Methods

### 2.1. Participants

Participants were recruited between September 2020 and April 2021 from four Spanish public secondary schools, one located in Madrid and three in Castilla-La Mancha, with a total of 1485 enrolled adolescents. Inclusion criteria for the adolescents were the following: (a) 12–18 years of age; (b) showing increased anxiety and/or depression symptoms based on the RCADS-30 [35] on at least one of its anxiety or depression subscales exceeding the following cut-off scores reported by Piqueras et al. [36]: major depressive disorder (MDD) = 4, panic disorder (PD) = 5, social phobia (SP) = 5, separation anxiety disorder (SAD) = 8, and generalized anxiety disorder (GAD) = 7; (c) having an e-mail address and daily internet access through a computer or tablet; and (d) Spanish proficiency. Exclusion criteria were the following: (a) a mental disorder diagnosis or a severe psychological problem (e.g., moderate or severe suicide risk); (b) ongoing psychological treatment; (c) a change in psychiatric medication dosage within the previous three months (if applicable); (d) a severe medical condition that would interfere with the study; or (e) no informed consent.

**Figure 1:**
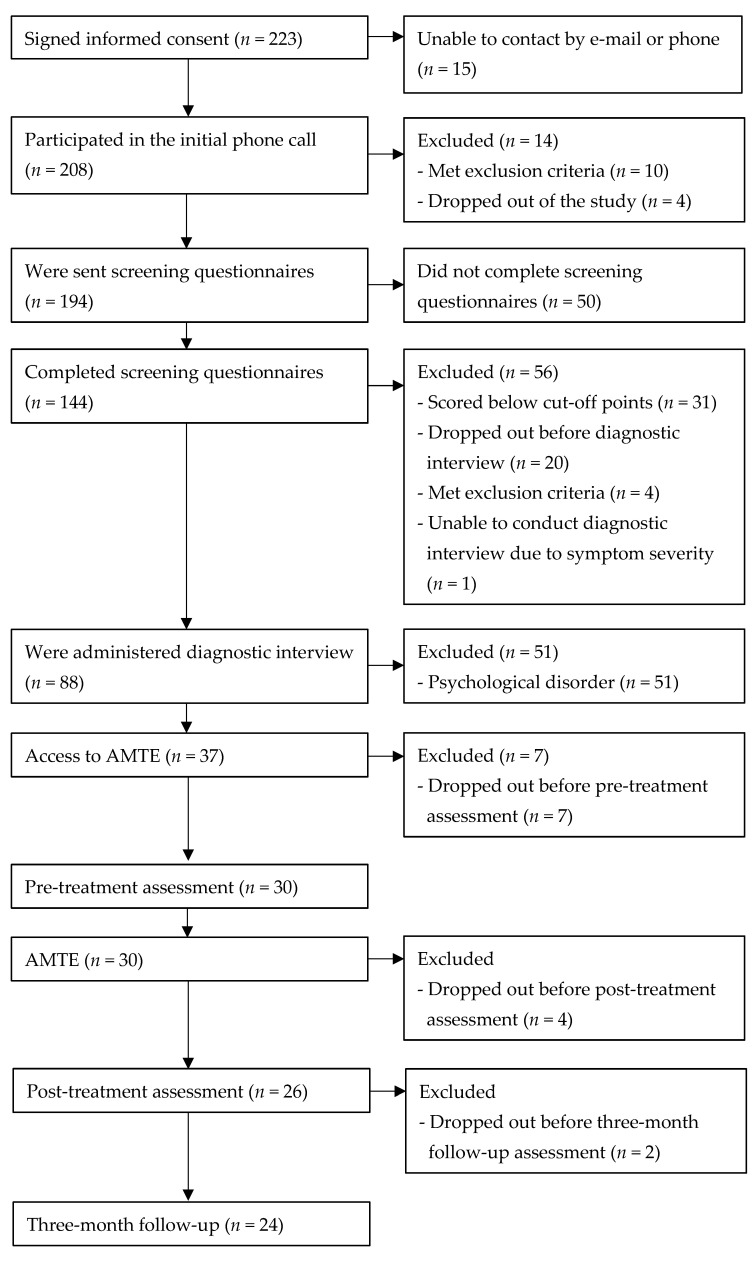
Participant flow through the study.

The total sample consisted of 30 adolescents (56.7% females, age range: 12–18 years, *M*_age_ = 14.00, *SD*_age_ = 1.89) who completed the pre-treatment assessment. Four adolescents dropped out before completing the post-treatment assessment and two before completing the three-month follow-up due to lack of time (*n* = 3) or other problems (*n* = 3). The completers (i.e., the participants who completed the program, the post-treatment, and the follow-up assessment) consisted of 24 adolescents (50% females, age range: 12–17 years, *M*_age_ = 13.50, *SD*_age_ = 1.47). See Figure 1 for the participant flow through the study.

### 2.2. Instruments

#### 2.2.1. Anxiety and Depression Measures

Mini International Neuropsychiatric Interview for Children and Adolescents (MINI-KID, version 1.1) [37]; Spanish version by Colón-Soto et al. [38]: Structured diagnostic interview that assesses the main disorders in children and adolescents according to DSM-IV and ICD-10. Sheehan et al. [39] found substantial to perfect test-retest and interrater reliabilities of ᴋ = 0.64–1.00 for all assessed disorders (including anxiety and depressive disorders). In the present study, psychologists conducted the interviews with the adolescents and interpreted their results.

Revised Child Anxiety and Depression Scale-30 (RCADS-30) [35]: Self-report questionnaire that assesses anxiety and depressive disorder symptoms in children and adolescents according to DSM-IV/5. For each of the 30 items, adolescents rated the frequency of the described event on a 4-point Likert scale from 0 (“never”) to 3 (“always”). The RCADS-30 includes the following subscales (5 items each): major depressive disorder (MDD), panic disorder (PD), social phobia (SP), separation anxiety disorder (SAD), generalized anxiety disorder (GAD), and obsessive-compulsive disorder (OCD). A total score, a total anxiety disorder score (ANX; summing all subscales except MDD), and a score for each of the six subscales can be computed. Piqueras et al. [40] showed an excellent internal consistency for the total scale and the total anxiety disorder scale (α = 0.93) and acceptable to good internal consistencies for each of the six subscales (α = 0.74–0.85).

Anxiety Scale for Children (Escala de Ansiedad para Niños, EAN) [41]: Self-report questionnaire that assesses general anxiety symptoms (physical, cognitive, and social) in children and adolescents. For each of its 10 items, adolescents rated the frequency of the described event on a 4-point Likert scale from 0 (“never or almost never”) to 3 (“many times or almost always”). The scale has good psychometric properties (α = 0.91–0.94) [18].

Depression Questionnaire for Children (Cuestionario de Depresión para Niños; CDN) [41]: Self-report questionnaire that assesses depression in children and adolescents through major depressive disorder and dysthymic disorder symptoms according to DSM-IV/5. For each of its 16 items, adolescents rated the frequency of the described event on a 3-point Likert scale from 0 (“never or almost never”) to 2 (“many times or almost always”). The scale has good psychometric properties (α = 0.87–0.89) [18].

PSWQ-11 questionnaire for children and adolescents (PSWQN-11) [42]: Self-report questionnaire that assesses pathological worry in children and adolescents. It is an age-downward version of the PSWQ-11 for adults [43]. For each of the 11 items, adolescents rated their agreement on a 5-point Likert scale from 1 (“totally disagree”) to 5 (“totally agree”). 

Clinical Global Impression Scale—Severity (CGI-S) [44]: Clinician-rated scale that assesses symptom severity. Clinicians rated the current symptom severity on a 7-point Likert scale from 1 (“normal, not at all ill”) to 7 (“extremely ill”). The scale is usually used in clinical populations and can be considered a valid instrument [45]. In the present study, we used the CGI-S to assess anxiety and depressive symptoms.

#### 2.2.2. Transdiagnostic Measures

Positive and Negative Affect Schedule for Children and Adolescents (Escalas PANAS para Niños y Adolescentes; PANASN) [46]: Self-report questionnaire that assesses positive and negative affect in children and adolescents. For each of the 20 items, adolescents rated the frequency of the described event on a 3-point Likert scale from 1 (“never”) to 3 (“many times”). Molina et al. [47] revealed good internal consistencies of α = 0.82 for the positive affect subscale (10 items) and α = 0.81 for the negative affect subscale (10 items). 

Childhood Anxiety Sensitivity Index (CASI) [48]; Spanish version by Sandín [49]: Self-report questionnaire that assesses anxiety sensitivity or discomfort with anxiety symptoms in children and adolescents. For each of its 18 items, adolescents rated the frequency of the described event on a 3-point Likert scale from 1 (“never”) to 3 (“many times”). The Spanish version has good psychometric properties (α = 0.89) [50]. 

Emotional Avoidance Strategy Inventory for Adolescents (EASI-A) [51]; Spanish version by García-Escalera et al. [52]: Self-report questionnaire that assesses emotional avoidance in adolescents. For each of its 17 items, adolescents rated the frequency of the described event on a 5-point Likert scale from 0 (“never or almost never”) to 4 (“always or almost always”). The total emotional avoidance scale reached a good internal consistency (α = 0.86) according to Kennedy and Ehrenreich-May [51].

#### 2.2.3. Reported Top Problems

Top Problems Assessment, adolescent version (TPA) [13], adapted from its original version by Weisz et al. [53]: During the first module of the program, adolescents and their parents were asked to define up to three top emotional problems afflicting the adolescent and related goals that they wanted to focus on during the program. Problems defined by parents could coincide with those defined by the adolescents or could differ. In each phone call, adolescents and their parents were asked to rate the severity of each problem on a 11-point Likert scale from 0 (“not at all severe”) to 10 (“very severe”). The TPA has shown good psychometric properties [53].

#### 2.2.4. Feasibility and Acceptability

Feasibility and Acceptability Questionnaire (FAQ) [54]. Self-report questionnaire that assesses experience with the online platform (6 items), satisfaction with the program (6 items) [55], and therapeutic alliance (6 items). There is an adolescent and parent version of the FAQ. The adolescents and their parents answered the questions put forward in each of the items on an 11-point Likert scale from 0 (“not at all”) to 10 (“totally” or “very much”). Parents also informed about their experience with the section for parents through two additional items: one dichotomous (yes/no) and the other rated on a 11-point Likert scale from 0 (“not at all”) to 10 (“totally”).

### 2.3. AMTE

Like the UP-A [14], AMTE uses evidence-based treatment techniques that cut across disorder-specific CBT treatment manuals for adolescent anxiety and depression, including psychoeducation, exposure, cognitive restructuring, behavioral activation, etc. It also includes motivational enhancement and mindfulness-based techniques. It includes similar modules as the UP-A, which are the following [32]: (1) Building motivation, (2) Getting to know your emotions, (3) Enjoy positive activities, (4) Awareness of your emotional experiences, (5) Learn to be flexible in your thinking, (6) Cope with your body sensations, (7) Cope with emotional situations, and (8) Maintain your gains. In this study, Modules 1 and 8 were delivered by a therapist via phone calls. Modules 2 to 7 are available on the web platform, and adolescents should do one module per week and spend one additional week working on Module 7, adding up to a total of 7 weeks. It takes about 30 min to complete a module on the platform. Each module consists of texts, videos, exercises, and assignments. To make the program more appealing, the design follows an island theme, the content is presented by a “doctor”, and participants receive motivational messages by an avatar they can design when they first access AMTE. A separate section for parents gives them information about their child’s progress and the possibility to download a summary of each completed module. The third section for therapists allows them to track participants’ progress, access the assignments, and register the weekly phone calls (described below). For more details about AMTE, see Sandín et al. [32,33].

### 2.4. Therapist Involvement via Phone Calls

Therapists involved in the study were psychologists or psychology students who had previously become familiar with the AMTE user platform, the section for parents and for therapists, and received a short training from the authors of the study. The psychology students received weekly supervision via video call or e-mail.

Adolescents and parents involved in the program received nine phone calls from a therapist. During all phone calls, the therapist talked separately with the adolescents and the parents. Modules 1 and 8 were delivered by the therapist during the first and last phone calls with the families. During the first phone call, up to three top problems they wanted to focus on during the program were defined, and motivation was built (Module 1). The adolescents then completed Modules 2 to 7 on the AMTE platform for 7 weeks, receiving a weekly telephone call from their therapist in which they revised the current module and its assignments and scored and commented on their top problems. Parents also scored and commented on the top problems and were given feedback on how to help their child with the top problems by applying strategies from AMTE (total weekly time per family for phone calls 1 to 8: approximately 20–30 min). During the ninth and last phone call with the adolescents, top problems were rated, and progress during the program was pointed out (taking into consideration possible setbacks during the program and in the future); core strategies learned in Modules 2 to 7 were summed up, emphasizing the strategies the adolescents found most helpful; and finally, plans were made on how to use these strategies in the future (Module 8). With parents, top problems were rated, and progress during the program was pointed out, taking into consideration possible setbacks during the program and in the future (total time per family for phone call 9: approximately 50 min). During a follow-up phone call, adolescents were given feedback on the follow-up questionnaire scores; they talked about how they were doing and how they were using the emotion regulation strategies learned during the program and rated the top problems. During a phone call with the parents, the same content was covered referring to their child (total time per family for the follow-up phone call: 30–50 min). 

### 2.5. Design and Procedure

For the present study, we used an uncontrolled pre-post design with a three-month follow-up. It was approved by the Research Ethics Committee of the Universidad Nacional de Educación a Distancia (UNED) and reported on Clinicaltrials.gov (last accessed on 30 March 2022) (identifier: NCT04182061).

The adolescents were referred to the researchers by school counselors who had detected potential emotional vulnerabilities, especially anxiety and depressive symptoms. School counselors provided the families with information about the study and collected informed consent. Referred adolescents and their families who were interested in participating in the study and had signed the informed consent were phoned by the researchers to establish the first contact, answer possible questions, and explain the following steps of the study to them. Exclusion criteria were assessed through this first phone call and the screening questionnaires. The latter could be accessed by the families via a link they received in an e-mail after the first phone call. The screening included a short first section for parents and a longer second section for adolescents. After completing the screening questionnaires, adolescents who showed increased self-reported anxiety and/or depressive symptoms and met the remaining inclusion criteria were invited to attend a diagnostic interview with one of the psychologists involved in the program. The psychologist also talked with one of their parents (or legal guardian) to obtain additional information about the adolescent’s mental health condition. The interviews were conducted via video calls. Adolescents who were diagnosed with a psychological disorder based on the interview were referred to mental health services or included in another study when they met its inclusion criteria. Adolescents who were not diagnosed with any psychological disorder were given access to AMTE, completed the pre-treatment assessment, and completed the program. During the last phone call, the therapists interviewed the adolescents and their parents about the feasibility and acceptability of the program and the platform. Finally, the adolescents completed the post-treatment assessment. After three months, they were asked to access AMTE once again to complete the three-month follow-up assessment. Subsequently, the adolescents and the parents received the follow-up phone call, during which they gave information about whether they had received psychological treatment, changed medication dosage for a psychological problem, or suffered a stressful event in the last three months. Therapists rated the CGI-S after the diagnostic interview, the ninth phone call, and the follow-up phone call. Families did not receive any incentives to participate in the study.

### 2.6. Statistical Analysis

Statistical analyses were performed using IBM SPSS Statistics for Windows, version 25.0 [56]. A significance level of α = 0.05 was applied. Completer analyses of participants who remained in the study until the follow-up (*n* = 24) and intention-to-treat analyses for the total sample (*n* = 30) were performed after replacing missing values for the dropout cases (*n* = 6) using the method “last observation carried forward” (LOCF). Exploratory analyses showed that there were some extreme outliers and that some variables were not distributed normally. For these reasons and the small sample size, we decided to conduct non-parametric statistical tests. Accordingly, we used Friedman tests to compare the different time points (pre-treatment, post-treatment, follow-up) for each variable relevant to our hypotheses. When Friedman tests were significant, we performed Wilcoxon signed-rank tests as post hoc tests to compare the different time points pairwise and corrected the exact one-tailed *p*-value according to Bonferroni (*p* × number of pairwise comparisons = *p* × 3) to control the family-wise error rate. Wilcoxon tests were also used to compare the ability to cope with emotions before and after the program according to the adolescents and their parents in the completer sample. Cohen’s d effect sizes and statistical power 1 − β were computed for each Wilcoxon test using G*Power [57]. Statistical power for significant Wilcoxon tests across the completer and total sample ranged from 1 − β = 0.37 to 1.00 (average = 0.82). Statistical power for non-significant Wilcoxon tests across the completer and total sample ranged from 1 − β = 0.05 to 0.78 (average = 0.28).

## 3. Results

### 3.1. Changes in Self-Reported and Clinician-Rated Anxiety and Depression Measures

See Table 1 for descriptive statistics at pre- and post-treatment and follow-up and Friedman tests for anxiety and depression measures. Completer and intention-to-treat analyses using Friedman tests (*df* = 2) revealed significant overall differences between the time points for general clinical depression symptoms (CDN), anxiety and depressive disorder symptoms (RCADS-30-total, MDD, PD, OCD, and ANX), and pathological worry (PSWQN-11). For general depressive symptoms (CDN), Wilcoxon tests showed a significant decrease from post-treatment to follow-up in the completer sample (*Z* = −3.05, *p* = 0.003, *d* = 0.42) and in the total sample (*Z* = −3.05, *p* = 0.003, *d* = 0.36). For anxiety and depressive disorder symptoms (RCADS-30-total), we found decreases from pre-treatment to follow-up (*Z* = −2.63, *p* = 0.009, *d* = 0.58) and from post-treatment to follow-up (*Z* = −2.82, *p* = 0.006, *d* = 0.36) in the completer sample and in the total sample (from pre-treatment to follow-up: *Z* = −2.27, *p* = 0.033, *d* = 0.43; from post-treatment to follow-up: *Z* = −2.82, *p* = 0.006, *d* = 0.28). For major depressive disorder symptoms (MDD), Wilcoxon tests revealed significant decreases from pre-treatment to follow-up (*Z* = −2.31, *p* = 0.027, *d* = 0.54) and from post-treatment to follow-up (*Z* = −2.17, *p* = 0.039, *d* = 0.32) in the completer sample and from post-treatment to follow-up in the total sample (*Z* = −2.17, *p* = 0.039, *d* = 0.27). For panic disorder symptoms (PD), we found a significant decrease from post-treatment to follow-up in the completer sample (*Z* = −3.24, *p* < 0.001, *d* = 0.49) and in the total sample (*Z* = −3.24, *p* < 0.001, *d* = 0.35). For obsessive-compulsive disorder symptoms (OCD), we observed significant decreases from pre-treatment to follow-up (*Z* = −2.71, *p* = 0.009, *d* = 0.50) and from post-treatment to follow-up (*Z* = −2.63, *p* = 0.009, *d* = 0.39) in the completer sample and in the total sample (from pre-treatment to follow-up: *Z* = −2.73, *p* = 0.006, *d* = 0.43; from post-treatment to follow-up: *Z* = −2.63, *p* = 0.009, *d* = 0.32). For total anxiety disorder symptoms (ANX), we found significant decreases from pre-treatment to follow-up (*Z* = −2.42, *p* = 0.021, *d* = 0.53) and from post-treatment to follow-up (*Z* = −2.47, *p* = 0.018, *d* = 0.33) in the completer sample and from post-treatment to follow-up in the total sample (*Z* = −2.47, *p* = 0.018, *d* = 0.25). For pathological worry (PSWQN-11), Wilcoxon tests revealed a significant decrease from pre-treatment to follow-up in the completer sample (*Z* = −2.99, *p* = 0.003, *d* = 0.87) and in the total sample (*Z* = −2.90, *p* = 0.003, *d* = 0.63). 

Friedman tests (*df* = 2) revealed significant overall differences between the time points for clinician-rated anxiety and depressive disorder symptom severity (CGI-S) in the completer and in the total sample. According to the results of the Wilcoxon tests, there were significant decreases from pre-treatment to post-treatment (*Z* = −3.47, *p* < 0.001, *d* = 1.22) and from pre-treatment to follow-up (*Z* = −3.51, *p* < 0.001, *d* = 1.28) in the completer sample as well as in the total sample (from pre-treatment to post-treatment: *Z* = −3.69, *p* < 0.001, *d* = 1.04; from pre-treatment to follow-up: *Z* = −3.74, *p* = < 0.001, *d* = 1.07). All Z-values were based on positive ranks.

### 3.2. Changes in Self-Reported Transdiagnostic Variables

See Table 2 for descriptive statistics at pre- and post-treatment and follow-up and Friedman tests for transdiagnostic measures. Completer and intention-to-treat analyses using Friedman tests (*df* = 2) revealed significant overall differences between the time points for emotional avoidance (EASI-A) and anxiety sensitivity (CASI) and a trend for positive affect in the total sample (PA; *p* = 0.071). For emotional avoidance, Wilcoxon tests showed a significant decrease from pre-treatment to follow-up in the completer sample (*Z* = −2.18, *p* = 0.042, *d* = 0.44) and no significant results in the total sample. For anxiety sensitivity, we found significant decreases from pre-treatment to follow-up (*Z* = −2.91, *p* = 0.003, *d* = 0.41) and from post-treatment to follow-up (*Z* = −2.51, *p* = 0.015, *d* = 0.35) in the completer sample and in the total sample (from pre-treatment to follow-up: *Z* = −2.46, *p* = 0.018, *d* = 0.27; from post-treatment to follow-up: *Z* = −2.51, *p* = 0.015, *d* = 0.26). All Z-values were based on positive ranks.

### 3.3. Changes in Self-Reported and Parent-Reported Top Problems Assessment Ratings

See Table 3 for descriptive statistics at pre- and post-treatment and follow-up and Friedman tests for the top problems. Completer and intention-to-treat analyses using Friedman tests (*df* = 2) revealed significant differences between the time points for all three top problems rated by the adolescents and the parents. For all three top problems rated by both the adolescents and the parents Wilcoxon tests showed significant decreases from pre- to post-treatment (Problem 1 adolescent: *Z* = −3.56, *p* < 0.001, *d* = 1.57; Problem 2 adolescent: *Z* = −3.87, *p* < 0.001, *d* = 1.55; Problem 3 adolescent: *Z* = −3.68, *p* < 0.001, *d* = 1.68; Problem 1 parent: *Z* = −3.31, *p* < 0.001, *d* = 1.62; Problem 2 parent: *Z* = −3.61, *p* < 0.001, *d* = 1.71; Problem 3 parent: *Z* = −3.44, *p* < 0.001, *d* = 1.65) and from pre-treatment to follow-up (Problem 1 adolescent: *Z* = −3.95, *p* < 0.001, *d* = 2.08; Problem 2 adolescent: *Z* = −3.56, *p* < 0.001, *d* = 1.63; Problem 3 adolescent: *Z* = −3.37, *p* < 0.001, *d* = 1.54; Problem 1 parent: *Z* = −3.64, *p* < 0.001, *d* = 1.81; Problem 2 parent: *Z* = −3.60, *p* < 0.001, *d* = 1.80; Problem 3 parent: *Z* = −3.36, *p* < 0.001, *d* = 1.38) in the completer sample, as well as in the total sample (from pre- to post-treatment: Problem 1 adolescent: *Z* = −3.93, *p* < 0.001, *d* = 1.52; Problem 2 adolescent: *Z* = −4.14, *p* < 0.001, *d* = 1.53; Problem 3 adolescent: *Z* = −3.88, *p* < 0.001, *d* = 1.43; Problem 1 parent: *Z* = −3.70, *p* < 0.001, *d* = 1.77; Problem 2 parent: *Z* = −3.94, *p* < 0.001, *d* = 1.65; Problem 3 parent: *Z* = −3.78, *p* < 0.001, *d* = 1.59; from pre-treatment to follow-up: Problem 1 adolescent: *Z* = −4.31, *p* < 0.001, *d* = 1.96; Problem 2 adolescent: *Z* = −3.85, *p* < 0.001, *d* = 1.59; Problem 3 adolescent: *Z* = −3.58, *p* < 0.001, *d* = 1.35; Problem 1 parent: *Z* = −3.96, *p* < 0.001, *d* = 1.94; Problem 2 parent: *Z* = −3.91, *p* < 0.001, *d* = 1.70; Problem 3 parent: *Z* = −3.67, *p* < 0.001, *d* = 1.37). All Z-values were based on positive ranks.

### 3.4. Self-Reported and Parent-Reported Feasibility and Acceptability

#### 3.4.1. Adolescent Report

In the completer sample (*n* = 24), participants completed between five and eight modules (*M* = 6.92, *SD* = 0.93), and the mean duration of weekly telephone calls (1 to 8) between the therapist and the adolescent was 15.55 min (*SD* = 4.59). In the total sample (*n* = 30), participants completed between one and eight modules (*M* = 6.30, *SD* = 1.66), and the mean duration of weekly telephone calls (1 to 8) between the therapist and the adolescent was 15.12 min (*SD* = 4.33).

Table 4 shows descriptive statistics for feasibility and acceptability rated by the adolescents in the completer sample (*n* = 24). Mean ratings for experience with the online platform ranged from 7.71 to 9.04 and for satisfaction with the program from 7.83 to 8.92 (first four items). Adolescents gained abilities to cope with emotions, with mean scores increasing from 4.88 (*SD* = 2.09) to 8.46 (*SD* = 1.10) comparing abilities from before and after the program (*Z* = −4.22, based on negative ranks, *p* < 0.001, *d* = 1.98). Mean ratings for therapeutic alliance were high and ranged from 9.04 to 9.67.

#### 3.4.2. Parent Report

In the completer sample (*n* = 24), the mean duration of weekly telephone calls (1 to 8) between the therapist and the parent was 12.51 min (*SD* = 4.90) and in the total sample (*n* = 30) 12.01 min (*SD* = 4.68).

See Table 5 for descriptive statistics for feasibility and acceptability rated by the parents in the completer sample (*n* = 24). Mean ratings for experience with the online platform ranged from 7.87 to 8.71. Fourteen parents logged in to the parent’s section of the AMTE platform and found it helpful, showing a mean rating of 8.08. Mean ratings for satisfaction with the program ranged from 7.75 to 9.58 (first four items). According to their parents, adolescents gained abilities to cope with emotions with mean scores increasing from 4.50 (*SD* = 2.72) to 8.50 (*SD* = 1.14) comparing abilities from before and after the program (*Z* = −4.30, based on negative ranks, *p* < 0.001, *d* = 1.69). Mean ratings for therapeutic alliance were high and ranged from 9.42 to 9.83.

## 4. Discussion

The development of transdiagnostic iCBT interventions is an emerging approach that allows delivering evidence-based treatments to adolescents who are presenting multiple comorbidities, including symptoms of anxiety and depression. To our knowledge, this is the first study on the utility of a prevention program for anxiety and depression in adolescents based on transdiagnostic iCBT. Overall, the results demonstrated that AMTE potentially had a significant effect on anxiety and depression in adolescents with subclinical symptomatology. In addition, the feasibility and acceptability of the online program ranged from good to excellent. These results are preliminary and should be interpreted with caution due to the small sample size and lack of a control group, which does not allow causal inferences and therefore the assessment of the efficacy of the program. Changes over time cannot be specifically attributed to the effects of the program. However, according to clinical and epidemiological studies, subclinical anxiety and depressive symptoms during adolescence (a time when these symptoms often start to emerge), rather than being temporary, tend to be chronic and worsen when left untreated [6,49,58,59,60,61,62,63]. Open trials such as the present study are of value in the early stages of a line of research, as they are cost-effective and reveal potential problems and opportunities for improvement for subsequent more costly studies.

Concerning the first hypothesis, we found significant improvements in anxiety and depressive symptoms from pre-treatment to follow-up and from post-treatment to follow-up. Specifically, there were significant reductions over time in general depressive symptoms (CDN); overall anxiety and depressive disorder symptoms (RCADS-30), overall anxiety disorder symptoms (ANX); MDD, PD, and OCD symptoms; and pathological worry. Regarding the magnitude of the changes, results showed small to medium within-group effect sizes (except for pathological worry, for which a large effect from pre-treatment to follow-up was found in the completer sample). These reductions in anxiety and depression at follow-up support the utility of AMTE for prevention purposes, which might be explained by its transdiagnostic nature (e.g., the adolescent learns new core strategies to deal with situations and cognitions that generate intense negative emotions) (see Sandín et al. [64]). In general terms, these results are in line with the ones previously reported by Sandín et al. [33] although the effect sizes are smaller (this could be explained by the fact that our previous study was mainly based on a clinical sample). 

We also found significant reductions in clinician-rated severity of anxiety and depressive symptoms from pre-treatment to follow-up. In line with our hypothesis, clinician-rated anxiety and depression severity decreased significantly from pre- to post-treatment and from pre-treatment to follow-up, showing large effects (both in the completer and in the total sample). The use of this measure is highly relevant, as it complements the information obtained through self-reported measures. The CGI-S has been used in several studies, e.g., [22,65], but, as far as we know, not yet in subclinical samples. The present study, therefore, represents the first one to use this scale in an adolescent population with increased anxiety and depressive symptoms but without a disorder diagnosis.

In line with our second hypothesis and in agreement with our previous work using AMTE in a sample of mainly clinical adolescents [33], in the present open trial, we found significant improvements with small effects on anxiety sensitivity (from pre-treatment to follow-up and from post-treatment to follow-up in the completer sample and the total sample) and emotional avoidance (from pre-treatment to follow-up in the completer sample). We also found a trend for positive affect in the total sample (*p* = 0.071). These results preliminarily suggest that AMTE was able to modify core transdiagnostic processes in subclinical adolescents, including anxiety sensitivity and emotional avoidance, and extend our previous findings obtained from a predominantly clinical sample [33]. These results also provide indirect evidence for the potential usefulness of AMTE as a selective prevention program.

Regarding the third hypothesis, we expected an improvement in the three main emotional problems (the top problems) rated by both the adolescents and the parents. All three top problems rated by the adolescents and their parents decreased significantly from pre-treatment to post-treatment and from pre-treatment to follow-up in the completer sample and the total sample showing large effects. We found bigger and more consistent effects than García-Escalera et al. [17], who also included top problems severity ratings using the UP-A as a universal preventive intervention. The decreases we found represent the improvement of real-life problems as perceived by the adolescents and their parents, providing evidence of high external validity. 

Finally, concerning feasibility, we found a low level of attrition (20%) compared with similar studies. This indicates that few adolescents abandoned the study compared to attrition rates found for other internet-/computer-based psychological treatments according to reviews; e.g., Melville et al. [66] found attrition rates ranging from 2 to 83% with a weighted average of 31%. However, only 33.3% of the participants completed all eight modules (the average number of completed modules was 6.92 out of the total 8 modules), which could be due to lack of time (possibly increasing the time available to do the program could facilitate completing the modules). Other outcome measures of feasibility assessed by means of the FAQ (experience with the online platform (usability) and therapeutic alliance) denote that AMTE is highly feasible both for adolescents and their parents, which is in line with results found in other studies evaluating the UP-A [17,18] and AMTE [33]. In addition, the satisfaction with the program ratings (acceptability) revealed that the adolescents and their parents were highly satisfied with AMTE. Both the adolescents and their parents reported significantly greater abilities of the adolescents in dealing with emotions after the program compared to before the program (large effects).

A strength of the present study is that it examined the first transdiagnostic internet-delivered intervention for the prevention of anxiety and depression in adolescents. The study provides preliminary evidence on the utility of AMTE for the indicated and, indirectly, for the selective prevention of anxiety and depression in adolescents. In addition, the findings are consistent with previous results of AMTE from a study with mainly clinical participants [33] as well as with the results of the UP-A reported by Ehrenreich-May’s group [13,22,67] and with the results of the UP-A adapted as a universal prevention program in Spain [17,18]. The scores obtained on the anxiety and depression measures were significantly reduced after the program, which is also consistent with the literature on the efficacy of transdiagnostic interventions based on the UP [12,68,69] and internet-delivered protocols [12,30]. Including different types of informants (multiple assessments), such as self-reports, parent reports, and clinician ratings, as well as a measure of real-life emotional problems (top problems) can also be considered a strength of the study. The use of these different variables allowed a more objective measurement of our intervention outcome. Likewise, the use of diagnostic interviews, unusual among psychological or psycho-educational anxiety and depression prevention studies for children and adolescents [10], and the inclusion of subclinical adolescents guaranteed the evaluation of AMTE for the indicated prevention of depression and anxiety. 

The main limitations of the present study are the lack of a comparison group and the small sample size. In addition, we only included a three-month follow-up, which is too short to show real prevention effects. Nevertheless, the significant effects at the three-month follow-up indicate a potential prevention effect (i.e., that AMTE might be more effective in the long-term than it is in the short-term). Although participants finished many modules, few participants finished all modules, which may have underestimated the effects of AMTE. 

Future studies should include a larger sample size to enable the use of parametric statistical analyses and increase power, longer follow-up periods to show a real prevention effect, active or passive control conditions to be able to clearly attribute effects to AMTE, and maybe diagnostic interviews at post-treatment and follow-up to rule out the presence of disorders and again show real prevention effects more clearly. Building motivation should be intensified, and program duration could be expanded to ensure that participants finish all modules so that we can clearly assess the effects of the program.

iCBT interventions such as AMTE have the potential to give adolescents access to quality treatment they might have difficulty to receive otherwise because of time, geographic or financial constraints, or stigma, among other things. They might also receive treatment faster, as waitlists for face-to-face interventions are usually long, which is especially relevant from a prevention perspective. All this becomes even more important in times of pandemics or similar circumstances that limit the availability of services (e.g., psychological interventions, transport), may be a financial and organizational burden on families, and require social distancing. As could be observed during the COVID-19 pandemic, in these times, the psychological burden on the whole population and especially on children and adolescents may be significant. iCBT interventions could be crucial to meet the associated higher need for psychological interventions, providing comparable effects on internalizing symptoms to face-to-face CBT interventions.

## 5. Conclusions

In this study, we tested the utility and feasibility of AMTE, a novel transdiagnostic iCBT intervention designed to manage anxiety and depression in adolescents, for prevention purposes. Despite the mentioned limitations, the study provides preliminary evidence in support of the potential utility of AMTE to reduce symptoms of anxiety and depression in adolescents with subclinical levels of internalizing symptomatology. Findings lend support to the utility of this program for the indicated prevention (and partially for the selective prevention) of anxiety and depressive symptoms and corresponding disorders. Therefore, AMTE could be applied to improve access to evidence-based CBT interventions for adolescents at risk of developing anxiety and/or depressive disorders and possibly other mental disorders related to these disorders. We have not only found improvements in comorbid symptoms of anxiety and depression but also reductions in symptoms of underlying transdiagnostic core etiological mechanisms of emotional disorders (i.e., anxiety sensitivity and emotional avoidance). Finally, the findings concerning feasibility (adherence, usability, and therapeutic alliance) and acceptability (satisfaction with the web platform) suggest that both the adolescents and the parents perceived the intervention as feasible and acceptable. Given the research gap concerning transdiagnostic prevention programs for adolescents, especially of an internet-delivered nature, the demonstration of the preliminary utility of AMTE seems of great relevance to begin to steadily close this gap.

## Figures and Tables

**Table 1:** Means (*M*) and standard deviations (*SD*) at pre- and post-treatment and follow-up and Friedman tests for anxiety and depression measures.

	Completer Sample Analyses *n* = 24								Intention-to-Treat Analyses *n* = 30							
Variables	Pre- Treatment		Post- Treatment		Follow- Up				Pre- Treatment		Post- Treatment		Follow- Up			
	*M*	*SD*	*M*	*SD*	*M*	*SD*	*χ*²	*p*	*M*	*SD*	*M*	*SD*	*M*	*SD*	*χ*²	*p*
EAN	7.63	4.50	6.50	4.34	5.75	5.10	4.42	0.110	7.43	4.55	6.63	4.45	6.03	5.08	4.15	0.126
CDN	11.04	7.31	11.58	8.59	8.00	8.54	10.57	0.005	11.10	6.65	11.80	7.91	8.93	8.07	9.92	0.007
RCADS-30																
Total	19.08	10.30	16.71	9.74	13.13	10.29	12.51	0.002	18.50	9.50	17.03	10.06	14.17	10.68	11.76	0.003
MDD	3.38	2.30	2.79	2.19	1.96	2.84	8.61	0.014	3.43	2.14	3.03	2.11	2.37	2.74	7.98	0.019
PD	1.00	1.38	1.79	2.50	0.71	1.60	11.89	0.003	0.93	1.41	1.77	2.69	0.90	2.11	11.51	0.003
SP	5.63	3.35	4.25	3.19	3.88	3.42	4.52	0.104	5.40	3.14	4.40	3.36	4.10	3.55	4.23	0.120
SAD	0.67	1.13	0.79	1.61	0.58	1.41	3.75	0.153	0.73	1.11	0.90	1.56	0.73	1.41	2.93	0.231
GAD	5.58	3.49	4.58	2.99	4.29	2.69	3.41	0.182	5.30	3.32	4.50	2.84	4.27	2.59	3.17	0.205
OCD	2.83	2.41	2.50	2.02	1.71	1.99	14.00	0.001	2.70	2.25	2.43	1.98	1.80	1.95	12.80	0.002
ANX	15.71	8.98	13.92	8.41	11.17	8.13	8.96	0.011	15.07	8.37	14.00	8.75	11.80	8.63	8.40	0.015
PSWQN-11	23.71	7.87	20.75	6.49	17.42	6.30	11.38	0.003	23.23	8.27	20.93	7.37	18.27	7.43	10.69	0.005
CGI-S	2.42	0.72	1.50	0.78	1.50	0.72	25.39	0.000	2.40	0.72	1.60	0.81	1.60	0.77	27.56	0.000

Note: ANX, total anxiety disorder symptoms (the five MDD symptoms were not computed for this measure); CDN, Depression Questionnaire for Children; CGI-S, Clinical Global Impression Scale—Severity; EAN, Anxiety Scale for Children; GAD, generalized anxiety disorder; MDD, major depressive disorder; OCD, obsessive-compulsive disorder; PD, panic disorder; PSWQN-11, Penn State Worry Questionnaire for Children and Adolescents-11; RCADS-30, Revised Child Anxiety and Depression Scale-30; SAD, separation anxiety disorder; SP, social phobia.

**Table 2:** Means (*M*) and standard deviations (*SD*) at pre- and post-treatment and follow-up and Friedman tests for transdiagnostic measures.

	Completer Sample Analyses *n* = 24								Intention-to-Treat Analyses *n* = 30							
Variables	Pre- Treatment		Post- Treatment		Follow- Up				Pre- Treatment		Post- Treatment		Follow- Up			
	*M*	*SD*	*M*	*SD*	*M*	*SD*	*χ*²	*p*	*M*	*SD*	*M*	*SD*	*M*	*SD*	*χ*²	*p*
PANASN																
PA	22.38	3.59	23.67	3.13	23.04	3.98	4.52	0.104	22.20	3.45	23.37	3.30	22.87	3.93	5.30	0.071
NA	16.88	2.64	16.42	2.86	15.63	3.45	2.93	0.231	16.87	2.73	16.60	3.02	15.97	3.53	2.36	0.307
EASI-A	24.92	11.42	22.50	11.22	19.75	12.25	7.68	0.021	26.07	12.34	24.47	12.40	22.27	13.53	7.22	0.027
CASI	25.63	5.99	25.17	5.35	23.33	5.27	11.03	0.004	25.90	5.59	25.80	5.68	24.33	5.84	10.26	0.006

Note: CASI, Childhood Anxiety Sensitivity Index; EASI-A, Emotional Avoidance Strategy Inventory for Adolescents; NA, negative affect; PA, positive affect; PANASN, Positive and Negative Affect Schedule for Children and Adolescents.

**Table 3:** Means (*M*) and standard deviations (*SD*) at pre- and post-treatment and follow-up and Friedman tests for top problems.

		Completer Sample Analyses *n* = 24									Intention-to-Treat Analyses *n* = 30							
Variables		Pre- Treatment		Post- Treatment		Follow- Up					Pre- Treatment		Post- Treatment		Follow- Up			
	*n*	*M*	*SD*	*M*	*SD*	*M*	*SD*	*χ*²	*p*	*n*	*M*	*SD*	*M*	*SD*	*M*	*SD*	*χ*²	*p*
Problem 1 (A)	22	5.68	1.62	2.64	2.15	2.23	1.69	23.46	0.000	26	5.50	1.61	2.62	2.08	2.27	1.69	30.54	0.000
Problem 2 (A)	22	6.00	1.93	2.77	2.22	2.45	2.36	25.33	0.000	26	5.96	1.93	2.85	2.13	2.58	2.27	27.02	0.000
Problem 3 (A)	22	6.05	1.96	2.68	2.06	2.27	2.76	19.75	0.000	26	6.23	2.12	3.04	2.34	2.69	2.94	19.85	0.000
Problem 1 (P)	20	6.15	2.21	2.85	1.81	2.25	2.10	25.49	0.000	24	6.21	2.19	2.67	1.74	2.17	1.95	31.01	0.000
Problem 2 (P)	19	6.53	2.20	2.89	2.05	2.37	2.41	23.06	0.000	23	6.65	2.04	3.04	2.31	2.61	2.61	24.70	0.000
Problem 3 (P)	18	6.11	2.22	2.61	2.00	2.56	2.81	20.13	0.000	22	6.32	2.08	2.82	2.30	2.77	2.91	21.78	0.000

Note: A, rated by the adolescent; P, rated by the parent.

**Table 4:** Descriptive statistics for feasibility and acceptability (FAQ) rated by the adolescents in the completer sample (*n* = 24).

**Experience with the Online Platform (Range: 0–10)**	***M***	***SD***
How easy has it been for you to use the AMTE online platform?	9.04	1.00
How easy has it been for you to understand what the videos and Dr. AMTE were telling you?	8.88	1.04
How useful has what you have been taught in the AMTE program (through the videos, the avatar, the PDFs, the homework assignments, etc.) been for you?	8.83	1.17
How easy has it been for you to include the AMTE program in your daily routine?	8.25	1.23
To what degree have you been able to do the exercises and homework assignments without technical or computer problems?	8.63	1.38
To what extent have you applied what you have learned from AMTE to your real life?	7.71	1.37
**Satisfaction with the Program (Range: 0–10)**	***M***	***SD***
How much have you learned from the program?	8.79	1.06
How effective has the program been in helping you cope with your problems?	8.50	0.98
How much have you enjoyed doing the program?	7.83	1.27
To what extent would you recommend the program to other adolescents?	8.92	1.10
How many skills to cope with emotions did you have before the program?	4.88	2.09
How many skills to cope with emotions do you have now?	8.46	1.10
**Therapeutic Alliance (Range: 0–10)**	***M***	***SD***
How much has your therapist helped you deal with your top problems?	9.04	0.91
How appreciated by your therapist have you felt?	9.50	0.78
To what extent have you felt that you and your therapist respected each other?	9.67	0.57
To what extent have you agreed with your therapist on what things were important for you to work on or overcome?	9.33	0.87
To what extent have you felt that your therapist cared about you?	9.63	0.65
How correct do you think the way you and your therapist have worked to solve your problems has been?	9.42	0.83

**Table 5:** Descriptive statistics for feasibility and acceptability (FAQ) rated by the parents in the completer sample (*n* = 24).

**Experience with the Online Platform (Range: 0–10)**	***M***	***SD***
How easy has it been for your son/daughter to use the AMTE online platform?	8.46	1.79
How easy has it been for your son/daughter to understand what the videos and Dr. AMTE were telling him/her?	8.37	2.10
How useful has what your son/daughter has been taught in the AMTE program (through the videos, the avatar, the PDFs, the homework assignments, etc.) been for him/her?	8.71	1.33
How easy has it been for your son/daughter to include the AMTE program in his/her daily routine?	7.87	1.75
To what degree has your son/daughter been able to do the exercises and homework assignments without technical or computer problems?	8.25	2.13
To what extent has your son/daughter applied what he/she has learned from AMTE to his/her real life?	8.25	1.57
**Experience with the parent’s section of the online platform**	***n* (%) (yes)**	
Have you ever logged in to the parent’s section?	14 (58.3%)	
To what extent have you found the parent’s section useful to help your son/daughter during treatment? (range: 0–10)	8.08	3.37
**Satisfaction with the program (range: 0–10)**	***M***	***SD***
How much has your son/daughter learned from the program?	8.79	1.14
How effective has the program been in helping your son/daughter cope with his/her problems?	8.71	1.40
How much has your son/daughter enjoyed doing the program?	7.75	1.80
To what extent would you recommend the program to other adolescents?	9.58	0.83
How many skills to cope with emotions did your son/daughter have before the program?	4.50	2.72
How many skills to cope with emotions does he/she have now?	8.50	1.14
**Therapeutic alliance (range: 0–10)**	***M***	***SD***
How much has the therapist helped your son/daughter deal with his/her top problems?	9.42	0.72
How appreciated by the therapist have you felt?	9.75	0.61
To what extent have you felt that you and the therapist respected each other?	9.79	0.59
To what extent have you agreed with the therapist on what things were important for your son/daughter to work on or to overcome?	9.83	0.48
To what extent have you felt that the therapist cared about your son/daughter?	9.75	0.53
How correct do you think the way they have worked to solve your son’s/daughter’s problems has been?	9.75	0.53

## Data Availability

Participants only agreed to share their data with the researchers involved in this study. Therefore, datasets are not publicly available. If you would like to get access, please contact B.S. (bsandin@psi.uned.es).
